# Timing Is Everything: Recognizing the Importance of Infusion Duration in Preoperative Antimicrobial Prophylaxis

**DOI:** 10.1017/ash.2024.179

**Published:** 2024-09-16

**Authors:** Peter Oakes, Lindsay Donohue, Sunny Chiao, Susan Ketcham, James Terenyi, Traci Hedrick, Amy Mathers

**Affiliations:** Fellow; UVA Health; University of Virginia

## Abstract

**Background:** For preoperative antimicrobials to be most effective in preventing surgical site infection, they must be administered early enough to reach a minimum tissue concentration that is specific to each drug. However, antibiotics have widely ranging infusion durations, from intravenous push over a few minutes to slow infusion over two hours. Heterogeneity in recommended infusion administration instructions, importance of infusion completion prior to incision, and complexity of healthcare systems present just some of the barriers to achieving appropriate preoperative antibiotic prophylaxis. We compared the percentage of infusion completion prior to case start before and after a multidisciplinary intervention. **Methods:** We performed a retrospective analysis of all patients undergoing a colorectal surgical procedure as defined by the National Healthcare Safety Network at a single university hospital from 10/19/22-10/18/23. A recognition that some antimicrobials were not finished infusing prior to surgery start prompted a multidisciplinary group including antibiotic stewardship, colorectal surgery, perioperative nursing, and anesthesiology to create and deploy an order set shortening metronidazole infusion duration from 60 to 30 minutes and initiating infusion in the preoperative area instead of the operating room. No change to the cefazolin intravenous push over 3-5 minutes was made. Goal antimicrobial infusion was defined as completed infusion within 120 minutes prior to incision, and calculations were made based on infusion start time and case start times. Rate of infusion completion was compared from the pre-intervention period to a post-intervention period from 10/19/23 through the end of the year. **Results:** For all colorectal surgeries in the pre-intervention period, 95% (n=418/440) of cefazolin doses and 0.002% (n=1/427) doses of metronidazole met goal infusion timing. At-goal infusion timing increased to 99% (n=84/85) of cefazolin doses and 68% (n=56/82) of metronidazole doses in the post-intervention period, resulting in a statistically significant improvement for metronidazole (Fischer’s exact test p < 0 .00001). The average time to metronidazole infusion completion changed from 45 minutes after procedure start to 58 minutes before procedure start. **Conclusions:** Multidisciplinary team engagement and deployment of an order set incorporating changes in duration and workflow for metronidazole infusion improved all antimicrobial preoperative infusions for colorectal procedures. Increased awareness of completing antimicrobial infusion prior to the incision may improve preoperative antimicrobial administration.

**Disclosure:** Lindsay Donohue: Advisor - Abbvie

